# Recent Progress in Obstetrics and Gynecology in Great Britain

**Published:** 1888-07

**Authors:** E. S. McKee

**Affiliations:** Cincinnati, Ohio


### RECENT PROGRESS IN OBSTETRICS AND GYNECOLOGY IN GREAT BRITAIN.

BY E. S. MCKEE, M. D., CICINNATI, OHIO.

Chlorosis, according to a recent statement of Sir Andrew Clarke, is a fecal anæmia. The symptoms of chlorosis he believes to be due to retention and absorption of the poisonous ptomains. Tonic aperients relieve this speedily and favorably.

The treatment of the pedicle in supra vaginal hysterectomy has been extensively studied by Dr. G. Granville Bantock, of the Samaritan Free Hospital for women, London. He treats it after the extra peritoneal method and reports a mortality of twelve out of seventy two. The number of septic causes from the intra-peritoneal method was greater than from the use of the extra-peritoneal. He thinks it a mistake to apply iron, or indeed anything to the stump. If properly trimmed it becomes as hard as horn in two or three days; the less the interference with the pedicle the better, if it has been rightly treated at the time of operation.

The surgery of the abdomen, and that of the brain, Dr. Robert Barnes considers epochs in surgery. He expresses himself as glad to know that they are British productions, and we are not indebted for them to the Germans.

Sterility, due to cervical endometritis, has been very successfully treated by Dr. Clement Godson in the out-patient department of St. Bartholomew's Hospital, by applications to the interior of the cervix of liq. ferri sulphatis and glycerine. This application promptly coagulates and removes the troublesome discharge and tends to its ultimate cure.

The proper treatment of anterior and posterior displacement of the uterus have received an impetus from the writings and practices of Macan, Master of the Rotunde Hospital, Dublin. This gentleman, an ardent follower of Schultse, and his views as to the real position of the uterus, has taught his British brethren that they must know first the normal position of the uterus before they can discuss its treatment when malpoised.

Meteorology, as an ætilogical factor in puerperal septicemia has been extensively discussed by Dr. Robert Barnes at the last meeting of the British Medical Association. He dwelt at length on the importance of pure air and removal of all impediments to a free circulation. A constant supply of air, dry and comparatively rare, as well as pure, is required. Damp, in the form of saturated air or mist, he considered to be the most injurious of all meteorological conditions. The lying-in chamber should face toward the south. A fire in an open fire-place will best combat the external damp. The open grate serves as a ventilator. From a sanitary point of view, heating by hot water pipes is abominable. The wind is the lowest, and meteorological conditions the worst in the early morning hours between two and four o'clock. The greatest care should be exercised as to the ventilation of the room about this time.

New theories are advanced by Dr. Berry Hart as to the mechanism of the delivery of the head in the ordinary forms of vertex presentation. From them he deduces a new method of protecting the perineum. He claims, the term distension of the head, as the fourth consecutive movement of the labor mechanism, is a most misleading one. He denies that the chin leaves the sternum while passing the perineum, and that during the anterior fixation of the pubic occiput under the pubic arch, anterior-posterior and increasing diameters of the fetal head form the ante-posterior diameter of the girdle of resistance. He thinks the best mechanism to avoid a tear, is for the occiput to lead, for the head to be driven on by the steady movement of translation, any rotation on a bi-parietal axis so taking place as to favor occipetal dipping, and never dipping of the sinciput.

The object is to restrain the head and also keep the occiput in the lead. To accomplish this, let the attendant press with his thumb, guarded by a napkin soaked within a hot sublimate solution, gently in front of the anus, the patient on her left side. This pressure should be made nearly in the direction of the pelvic outlet. This prevents the descent of the sinciput, and favors that of the occiput.

Embryotomy has been used so infrequently of recent years that the hope has been entertained that in the not far distant future that such instruments as the crochet, cephalatribe, and craniotomy forceps, will be relegated from the obstetric armamentarium to the chamber of horrors of some future museum of surgical instruments. The greater frequency with which forceps are used has had much to do with lessening the proportion of craniotomy cases. The mortality of forcep cases has decreased as their numbers have increased.

Expression of the placenta, a method generally accredited to the genius of Crede, is stoutly claimed by the Dublin school. Dr. Macan, in his Presidential Address, before the Section in Obstetrics of the British Medical Association, at Dublin, in August last, argued this point in an able manner. He showed the ward book of Dr. Charles Johnson, of the Rotunda, Hospital, bearing the date 1848. After numerous cases there is the terse statement, "placenta pressed out." This practice was so common as to need no further comment. He had discovered the curious fact that while in Germany, we have the highest authorities, Spiedelberg and Schroeder, favoring the Dublin method, in England the writers of the text-books recommend, or rather he would say, while describing and recommending the Dublin system, they call it Crede's.

Nymphomania has been made the subject of much study by Dr. Routh, of London. An important point which he brings out well, is that these women are liars; plausible liars, cunning liars—liars not necessarily from ill will, or even free will, but from disease. They are a class of patients with whom the reputation of no medical man is safe, and against whom we cannot be too frequently warned.

External palpation of the pregnant uterus is carried to such perfection at the Rotunda Hospital that they often feel the child's arm going out from the shoulder, while occasionally the elbow can be made out. In one case a hydrocephalic head was readily distinguished; in another the probability of the fœtus being an encephalic was foretold, while in one case the cord has been felt crossing the child's back, and quite recently they have thought they could make out the placental situ by the fingers being lifted off the fœtus and the outlines developed. Cases in which the placenta can be palpated will always be rare. To make this possible, the placenta must be situated on anterior abdominal walls and the other conditions for palpation be favorable.

Gonorrhœa in women is the subject of a lengthy discussion by Dr. W. Cinclair, of Manchester. This gentleman advances the claim that there is no vaginal gonorrhœa, but that the disease attacks the uterus from the start. In examining the discharges for the gonoccus, specimens should be taken from the urethra, the neck of the uterus, and in exceptional cases from the orifices of the glands of Bartholini, and the cast should be as recent as possible. He dwells at length on the dangers which may result to women from latent gonorrhœa in the h :sband. He thinks the disease may remain latent in the husband until evoked by excesses after marriage. It may also remain latent in women, he thinks, and be transferred to the second husband after marriage.

The question of antisepsis is nowhere discussed so bitterly as in London. Almost every hospital has its advocates for and opponents against this subject. At the Samaritan Free Hospital for women and children, Mr. J. Knowsley Thornton opeıates under the spray, and with all the orthodox antiseptic precautions. In the same hospital Dr. Granville Bantock operates with no regard whatever to antisepsis, and in many cases flushing out the perineum with plain, simple water, containing each and every one ot of its "38 beasts." Watching results, we find those of the one about equal to those of the other.

